# Pyelo-ureteral junction obstruction in poorly functioning kidneys: Does conservative management play a role in pediatric patients?

**DOI:** 10.3389/fped.2023.1108170

**Published:** 2023-03-22

**Authors:** Angelo Zarfati, Ermelinda Mele, Maria Felicia Villani, Nicola Capozza, Marco Castagnetti

**Affiliations:** ^1^ Pediatric Urology Unit, Bambino Gesù Children’s Hospital, IRCCS, Rome, Italy; ^2^ Department of Pediatric Surgery, Bambino Gesù Children’s Hospital, IRCCS, Rome, Italy; ^3^ University of Rome Tor Vergata, Rome, Italy; ^4^ Department of Imaging, Nuclear Medicine Unit, Bambino Gesù Children’s Hospital, IRCCS, Rome, Italy; ^5^ Department of Surgery, Oncology, and Gastroenterology, University of Padova, Padua, Italy

**Keywords:** pyelo-ureteral junction obstruction, conservative treatment, surgery, nephrectomy, pyeloplasty, pediatric, poorly functioning kidneys, hydronephrosis

## Abstract

**Background:**

Management of Pyelo-ureteral Junction Obstruction (PUJO) in poorly functioning kidneys in pediatric patients is still controversial, particularly regarding the role of conservative treatment.

**Aim:**

To evaluate and present the outcomes of internal diversion and follow-up results of a small series of pediatric patients with UPJO in poorly functioning kidneys.

**Study design:**

Retrospective review of 17 consecutive patients with unilateral PUJO in kidneys with Differential Renal Function (DRF) <20% undergoing temporary internal urinary diversion between 2009 and 2021 at a single tertiary center. DRF was reassessed after 1–3 months of diversion and subsequent management was conservative or surgical (pyeloplasty or nephrectomy) based on surgeon’s and family’s preferences without randomization.

**Results:**

After a trial of internal urinary diversion, 4/17 patients (23%) showed a DRF increase ≥5% (9%–12%), up to a maximum DRF of 28%, 3 underwent pyeloplasty, while 1 was managed conservatively. The remaining 13 patients showed no differential renal function improvement after diversion, and 7 were managed expectantly while 6 surgically (4 pyeloplasty, 2 nephrectomy). Overall, nine patients (53%) were managed surgically and 8 (47%) expectantly After a median (range) follow-up of 3.1 (0.3–7.9) years, no significant difference was observed between groups regarding symptoms (*p* = 0.205), need for further surgery (*p* = 1.000), and renal function (*p* = 1.000).

**Discussion:**

Although fraught with the limitation of a small sample size, this is the first study reporting on the conservative management of this controversial group of patients.

**Conclusion:**

In present pediatric series of pyelo-ureteral Junction obstruction in poorly functioning kidneys with differential renal function <20%, function recovery after a trial of internal urinary diversion was quite exceptional, and no difference was observed in outcome between patients managed surgically and conservatively after stent removal.

## Introduction

In a patient with Pyelo-Ureteral Junction Obstruction (PUJO) like hydronephrosis, the presence of renal function impairment, defined as a differential renal function (DRF) on the affected side <40% is generally considered an indication for surgery (1). Nevertheless, some patients present with a severely impaired DRF, which cut-off is quite variable and arbitrary in the literature generally ranging between <20% and <10% (2–7). Under these circumstances, management becomes more controversial. Some authors recommend direct nephrectomy, others direct pyeloplasty, and others a trial of temporary urinary diversion to begin with in order to assess the actual potential for function recovery before embarking on either nephrectomy or pyeloplasty (2–7). The evidence, however, indicates that the likelihood of renal function recovery is generally limited in these situations and that no factor can reliably predict it (2). Additionally, although conservative management is the first line treatment in patients with PUJO-like hydronephrosis and normal DRF (8, 9), to our knowledge, no pediatric series exists of patients with PUJO in poorly functioning kidneys treated nonoperatively.

The purpose of this study was to report the outcomes of internal diversion and follow-up results of a small series of pediatric patients with UPJO in poorly functioning kidneys comparing those treated operatively to those managed conservatively. Our hypotheses were that diversion hardly allows for function recovery and conservative management might be a viable option.

## Methods

A retrospective review of consecutive patients with PUJO in poorly functioning kidney treated with a temporary urinary diversion at single tertiary center between 2009 and 2021 was undertaken. Inclusion criteria were age <18 years, unilateral PUJO, and ipsilateral Differential Renal Function (DRF) <20%. The cut-off of DRF <20% was arbitrary, but consistent with previous studies (2). Exclusion criteria were incomplete follow-up data, secondary PUJO, or associated upper and/or lower urinary tract anomalies. The number of cases in the area during the study period determined the sample size. Locally, nationally, and internationally referred patients composed the study population. Clinical, radiological, and surgical variables were revised.

PUJO was diagnosed in patients with persistent/worsening hydronephrosis on ultrasound and decreased DRF on diuretic renography. At the outset, the patients were evaluated with a diuretic renogram to confirm the obstruction and assess the DRF. Patients underwent a temporary internal urinary diversion with a Double-J stent (DJ). For the purpose of this study, the images of the pyelography performed at stent placement were reviewed by two of the authors and the cause of PUJ was classified as intrinsic (ureter entering the pelvic straight in a dependent position) or kinking (such as in case of high insertion or suspicion of extrinsic obstruction). A diuretic renogram was obtained with diversion in place after 1–3 months to assess any function recovery. In the absence of a standardized DRF cut-off on diuretic renography, we defined as significant improvement any increase in DRF ≥5%. Treatment options included removal of the ureteral stent and conservative management, pyeloplasty (laparoscopic or laparo-assisted dismembered Anderson-Hynes pyeloplasty), or nephrectomy (retroperitoneoscopic). There was no randomization. Patients were followed-up with serial clinical and sonographic checks at increasing intervals (usually at 2–6–12 months and yearly thereafter). A diuretic renogram was obtained during subsequent follow-up only in patients developing symptoms or with worsening hydronephrosis.

Primary endpoints to compare patients managed surgically vs. expectantly included symptoms (e.g., pain, hypertension, UTIs, etc.), need for further surgery, and renal function. The latter was defined as normal if serum creatinine was within normal range for age and weight, and there was no hypertension requiring treatment.

Categorical variables were reported as absolute and relative frequencies (%). Continuous variables are reported as median and range. Groups were compared using the Chi Square test or the Fisher exact test for categorical variables, as appropriate. For continuous variables, differences between groups were established with a non-parametric test, *U* Mann–Whitney test. All *p*-values were two-sided, and a value <0.05 was considered significant.

This study did not receive any specific grant from funding agencies in the public, commercial, or for nonprofit sectors. The authors have no financial relationships or conflicts of interest to disclose.

The present study received authorization for publication from the scientific board in the authors’ institution.

## Results

**Figure 1:**
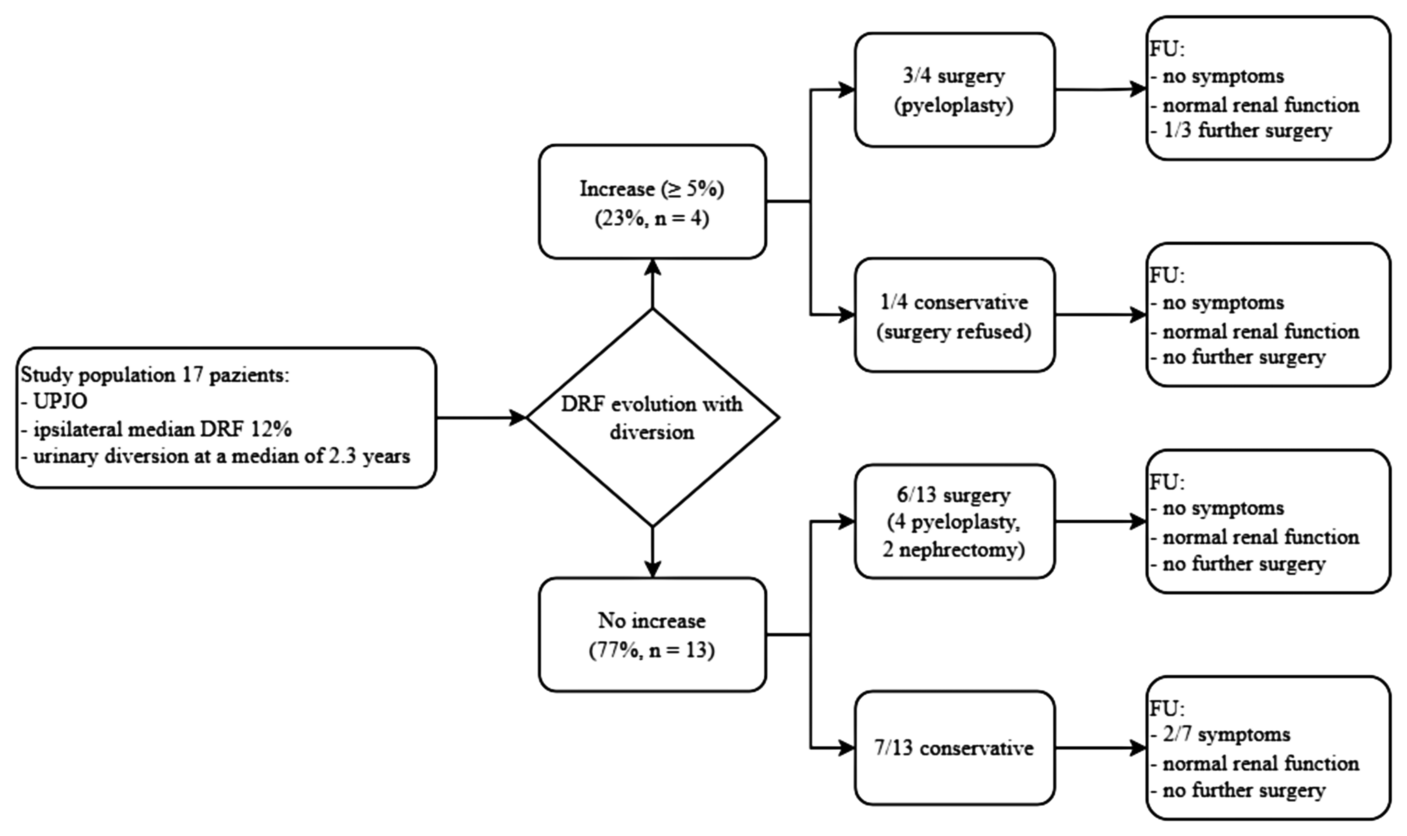
Management, follow-up, and outcomes according to evolution of DRF with urinary diversion. UPJO, Ureteropelvic Junction Obstruction; DRF, Differential Renal Function.

The series included 17 patients (Tables 1, 2 and Figure 1). After DJ insertion, 4/17 (23%) patients showed a DRF increase ≥5% (median 11.5%, range 9%–12%), for a final maximum ipsilateral DRF of 28% in one patient. Of note, age of these 4 patients was 2.1, 2.3, 7.4, and 14 years, whereas none of the 6 patients undergoing diversion in the first 18 months of life showed any significant DRF improvement. DFR improvement occurred in 3 out of 8 patients with evidence of an intrinsic obstruction on pyelography vs. 1/6 with evidence of ureteral kinking. Two of the kidneys with DRF less than 15% experienced an increase of DRF ≥5% with diversion (Table 2).

**Table 1:** Population, diagnosis and management, and follow-up.

Population	17
Male *n* (%)	14 (82%)
Prenatal diagnosis *n* (%)	12 (70%)
Symptoms *n* (%)	Total	6 (35%)
	*Flank/abdominal pain*	*4*
	*UTIs*	*1*
	*Water-electrolyte imbalance*	*1*
Normal renal function *n* (%)	17 (100%)
Left side *n* (%)	10 (59%)
AP-axis Median (range) mm	27 (14–63)
DRF at diagnosis Median (range) %	12 (1–19)
Age at diversion Median (range) years	2.3 (0.4–16.6)
Days of diversion Median (range)	64 (28–392)
DRF with diversion Median (range) %	13 (0–28)
Increase of DRF ≥5% with diversion *n* (%)	4 (23%)
Median increase with diversion (in patients with ≥5%) Median (range) %	11.5% (9%–12%)
Management *n* (%)	Conservative	8 (47%)
	Surgery	Total	9 (53%)
		*Pyeloplasty*	*7*
		*Nephrectomy*	*2*
FU since diversion Median (range) years	3.1 (0.3–12.4)
Age at FU end Median (range) years	6.2 (1.6–18.6)

UTIs, urinary tract infections; AP, anteroposterior; DRF, differential renal function; FU, follow-up.

**Table 2:** Individual details of the patients.

Patient	DRF at diagnosis (%)	Age at diversion (y)	PUJ anatomy at pyelography	DRF with diversion (%)	Increase with diversion (%)	Management	Symptoms during the FU	Further surgery during the FU
1	14	2.3	Intrinsic obstruction	13	No	Pyeloplasty	No	No
2	12	1.0	Kinking	11	No	Conservative	No	No
3	7	7.4	Intrinsic obstruction	15	+8	Pyeloplasty	No	No
4	18	15.8	Kinking	17	No	Conservative	No	No
5	0	4,3	Kinking	0	No	Conservative	No	No
6	7	2.3	Intrinsic obstruction	7	No	Conservative	Yes	No
7	13	2.9	Not available	17	+4	Pyeloplasty	No	No
8	14	2.4	Intrinsic obstruction	11	No	Conservative	No	No
9	16	14.0	Kinking	28	+12	Pyeloplasty	No	Yes
10	10	1.7	Intrinsic obstruction	9	No	Conservative	No	No
11	3	0.5	Kinking	0	No	Nephrectomy	No	No
12	15	2.3	Intrinsic obstruction	26	+11	Conservative	No	No
13	3	1.4	Intrinsic obstruction	2	No	Conservative	Yes	No
14	19	16.6	Not available	19	No	Pyeloplasty	No	No
15	5	0.4	Not available	6	+1	Nephrectomy	No	No
16	15	0.5	Kinking	19	+4	Pyeloplasty	No	No
17	12	2.1	Intrinsic obstruction	24	+12	Pyeloplasty	No	No

Of the 4 patients showing a DRF improvement >5% after diversion, 3 underwent pyeloplasty, while 1 was managed conservatively for parental preference. The remaining 13 patients showed no DRF improvement after diversion, and 7 were managed expectantly while 6 surgically (4 pyeloplasty, 2 nephrectomy).

Overall, of 17 patients, 9 (53%) were managed surgically (7 pyeloplasty, 2 nephrectomy) and 8 (47%) expectantly. Median (range) follow-up after diversion was 3.1 (0.3–12.4) years and median (range) age at last follow-up was 6.2 (1.6–18.6) years. Outcomes of patients undergoing operative vs. nonoperative management are detailed in (Table 3). No statistically significant difference was observed regarding symptoms (*p* = 0.2), need for further surgery (*p* = 1.0), renal function (*p* = 1.0), despite a comparable length of follow-up (*p* = 0.9) and age at last follow-up (*p* = 0.3). No surgically treated patients developed symptoms, but one (11%) underwent an endoscopic balloon dilatation of the pyelo-ureteral anastomosis to rule out a recurrent obstruction after a pyeloplasty. Two patients (25%) managed conservatively developed symptoms during follow-up, a mild occasional hypertension which did not require any treatment and a single episode of urosepsis treated medically, respectively. None of the patients managed conservatively underwent further surgeries. All the patients had normal renal function at last follow-up.

**Table 3:** Surgery and conservative management follow-up outcomes.

	Total	*Conservative*	*Surgery*	*p* value
	(*n* = 17)	(*n* = 8, 47%)	(*n* = 9, 53%)	
Symptoms *n* (%)	2 (11%)	2 (25%)	0 (0%)	0.205
Further surgery *n* (%)	1 (5%)	0 (0%)	1 (11%)	1.000
Normal renal function *n* (%)	17 (100%)	8 (100%)	9 (100%)	1.000
FU Length Median (Range) *y*	3.1 (0.3 12.4)	3.5 (1.9–12.4)	2.0 (0.6–11.0)	0.888
Age At FU End Median (Range) Y	6.2 (1.6–18.6)	6.8 (3.6–18.6)	6.2 (1.6–18.5)	0.312

FU, follow-up.

Table 4 summarizes results according to the changes in DFR during diversion. Of the 4 patients showing DRF improvement (3 pyeloplasty and 1 managed conservatively), none developed symptoms, one underwent a balloon dilatation of the pyelo-ureteral anastomosis, and all had normal renal function at last follow-up. Of the 13 patients with no increase of the DRF (7 managed expectantly, 4 pyeloplasty, and 2 nephrectomy), 2 managed conservatively developed symptoms during follow-up (1 occasional hypertension, 1 single episode of urosepsis), none underwent further surgery, and all had normal renal function at last follow-up.

**Table 4:** Management and follow-up and outcomes according to evolution of differential renal function with diversion.

		Total	*DRF increase*	*No DRF increase*
		(*n* = 17)	(*n* = 4)	(*n* = 13)
Conservative *n* (%)	8 (47%)	1 (25%)	7 (54%)
Surgery *n* (%)	Total	9 (53%)	3 (75%)	6 (46%)
	Pyeloplasty	7	3	4
	Nephrectomy	2	–	2
Symptoms *n* (%)	2 (11%)	0 (0%)	2 (15%)
Further surgery *n* (%)	1 (5%)	1 (25%)	0 (0%)
Normal renal function *n* (%)	17 (100%)	4 (100%)	13 (100%)

DRF, differential renal function; UTIs, urinary tract infections; US, ultrasound; FU, follow-up.

## Discussion

In our experience, the chances of recovery of poorly functioning, obstructed kidneys was low, only 4 patients experienced an increase of DRF ≥5% and maximum increase was 12%. Patients managed surgically and expectantly had similar outcomes in terms of symptoms, need for further surgery, and final renal function.

A trial of urinary diversion either external with a percutaneous, nephrostomy, or internal with a DJ stent, as in current series, is theoretically rational under these circumstances to assess function salvageability and choose the most appropriate treatment. Such a strategy was initially popularized in the 1980s (10–15). However, reported outcomes in recent series are quite inconsistent, with improvements in renal function following diversion seen in 24%–100% of patients. Such variability can perhaps be accounted for by the heterogeneity of studies particularly in terms of the cut-offs used to define poor function (DRF <20%, <15%, or <10%) (3–6, 16), and significant DRF improvement (DRF ≥10% at control (6, 16); DRF increase ≥10% compared to the baseline (5); DRF ≥10% at control and PCN drainage greater than 200 ml per day (4); SRF and GFR more than 10% (3)), respectively. Unfortunately, all these parameters remain somewhat arbitrary. The results of present series seem to support the principle that function recovery is rare and often limited. Additionally, no criteria can predict which patients will experience function recovery (17, 18). Consistently, in contrast with common sense, none of the patients undergoing diversion below 18 months experienced significant function recovery, whereas the patient experiencing the greatest degree of function recovery was one of the oldest of the series. A recent systematic review suggested that renal function recoverability might be more likely in patients with a DRR at the outset <15% (18). We had no many patents with a DRF in the range 15%–20%, but function recovery occurred both in patient with a DRF at the outset above and below this cutoff (DRF 7%, 12%, 15%, 16%, respectively. Table 4). Overall, after reviewing our experience, we now question the role of a trial of urinary diversion in patients with poorly functioning, obstructed kidneys. Given the limited chances of function recovery, the costs and risks of two procedures and two anesthesia, in our opinion, are not warranted. Moreover, similar considerations apply also to an external diversion. Although the latter can potentially be placed and removed without anesthesia, it is perhaps more bothersome for the patients and their family. Previous studies suggested direct pyeloplasty as a viable alternative for those families wishing to make any attempt to preserve renal function, but double J stent placement and removal seem quicker and simpler procedures (19).

Beyond the debate surrounding urinary diversion, conservative treatment, nephrectomy, or pyeloplasty can all be used as final management. The first is often claimed as an option, but, in fact, to the best our knowledge, this is the first series reporting results. Interestingly enough, outcomes appeared to be pretty comparable between patients managed operatively and nonoperatively. In keeping with previous series (2), present one confirms that nephrectomy is the approach with the lowest risk of long-term issues and therefore the lowest need for long-term follow-up. Consistently, in retrospect, we believe that our choice to perform a pyeloplasty in the 4 patients with no increase in DRF after diversion, was a mistake and we would no longer offer this option in this scenario.

The results of present experience must be qualified by its retrospective nature and the extremely limited sample. The main limit of the study is the lack of randomization. Moreover, due to our methodology, we may have missed patients for whom conservative treatment without a trial of urinary diversion was elected during the study period. Furthermore, owing to the lack of a standardized prospective protocol for management of pediatric patients with PUJO in poorly functioning kidneys, decisions were left to clinicians’ and family/caregivers’ discretion. Therefore, larger, prospective, multicenter studies are required to validate our results.

## Conclusions

Even if the numbers within the study are too small to draw any definitive conclusion, in our experience, only one-fourth of cases experienced an improvement in differential renal function >5%. Outcomes were not significantly different between patients managed conservatively and operatively after stent removal.

## Data Availability

The raw data supporting the conclusions of this article will be made available by the authors, without undue reservation.
